# Assessment of typing methods, virulence genes profile and antimicrobial susceptibility for clinical isolates of *Proteus mirabilis*

**DOI:** 10.1186/s12941-024-00770-8

**Published:** 2025-01-15

**Authors:** Lamiaa A. Salama, Hazem Hamed Saleh, Shaymaa H. Abdel-Rhman, Rasha Barwa, Ramadan Hassan

**Affiliations:** 1 Present Address: Microbiology and Immunology Department, Faculty of Pharmacy, Mansoura University, Mansoura, 35516 Egypt; 2 Urology and Nephrology Center, Mansoura University, Mansoura, Egypt; 3 Microbiology and Immunology Department, Faculty of Pharmacy, Horus University, New Damietta, 34518 Egypt

**Keywords:** *P. mirabilis*, Antimicrobial resistance, Virulence, PCR-based molecular typing, Discrimination power, Congruence

## Abstract

*Proteus mirabilis* (*P. mirabilis*) is one of the most important causative pathogens associated with complicated urinary tract infections with a 20% incidence. For epidemiological determinations, several phenotypic and molecular typing methods have been implicated. Sixty *P. mirabilis* isolated undergo antibiotic susceptibility test by standard Kirby Bauer method. They showed high resistance to nitrofurantoin and trimethoprim/sulfamethoxazole that appear mainly in 3rd age group. The 2nd age group comprised most of the resistant isolates to the tested antibiotics. A total of 73.33% of isolates were classified as multi drug resistance (MDR) and 78.3% of isolates were distributed in several antibiotypes with MAR index over 0.2. Twenty-one isolates were strong biofilm-producers and they were significantly related to MDR. Different virulence factors as protease, urease and hemolysin production are detected. Detection of several virulence genes by PCR; *zapA* and *ureC* were harbored by all isolates, followed by *rsbA* (95%), *ureA* and *flaA* (93%), *hpmA* (91.7%) and *mrpA* (73.3%). Determination of genetic diversity between isolates was performed by different methods (RAPD, ISSR, ERIC, BOX-AIR and REP-PCR) by using several parameters as typeability and discriminatory power indicating that ERIC-PCR was the best method followed by REP-PCR 1R. Rand’s & Wallace coefficients were used for calculating the congruence among typing methods. Conclusions: The results obtained from both conventional and molecular typing methods indicated that molecular methods are superior to conventional methods in the discrimination of isolates. ERIC-PCR and Rep-PCR provide high discrimination ability among *P. mirabilis* clinical isolates contributing to epidemiological studies.

**Supplementary Information:**

The online version contains supplementary material available at 10.1186/s12941-024-00770-8.

## Introduction

*Proteus* species (spp.) are distributed worldwide. They can be detected in wastewater, soil, and dung, where they aid in the degradation of animal-derived organic matter [1]. *Proteus mirabilis* is a Gram-negative rod-shaped bacterium characterized by its swarming motility and urease function, and it is often responsible for catheter-associated urinary tract infections (CAUTI) [2]. These infections can be followed by urolithiasis, the formation of bladder or kidney stones as a result of the alkalization of urine pH caused by the release of ammonia from urea hydrolysis by urease enzyme, which induces calcium and magnesium crystallization, and obstructing the lumen of indwelling catheters [3, 4].

The widespread use of antibiotics contributes to the spread of antimicrobial drug resistance and is crucial for selecting microorganisms with resistance genes. The World Health Organization considers this to be the most important problem. In various areas of the world, *P. mirabilis* has been associated with numerous nosocomial infection outbreaks and community-acquired infections. The most often prescribed groups for life-threatening nosocomial infections are 3rd generation cephalosporins, fluoroquinolones, and aminoglycosides. Finally, some studies have shown that *Enterobacteriaceae*, including *P. mirabilis*, are highly resistant to these antibiotic groups [5].

Swarming motility, fimbriae, urease, hemolysin, protease, and lipopolysaccharides are some of the virulence factors found in *P. mirabilis*. It can colonize, live, and grow in the host thanks to these virulence factors. In addition, it can create biofilms that colonize the airways and resist antibiotic therapy [1]. During swarming, virulent genes encoded in operons synergistically regulate the expression of several of these virulence factors. The ability to swarm on solid surfaces is one of the most prominent properties of *Proteus* spp.; even though several genes are associated with the swarming phenomenon like *cheW*, *cldA* and *gidA* genes. In addition, the most important gene for regulating the swarming is *rsbA* gene [6, 7].

Urease enzyme is the most essential enzyme for kidney and bladder stone development in *Proteus* infection. Urease enzyme is expressed by the gene’s *ureA*, *ureB*, *ureC*, *ureD*, *ureE*, *ureF*, *ureG*, and *ureR* on the *ure* operon. Studies have documented *ureA*, and *ureC* as major genes for urease production. Protease enzyme is encoded by *zapA*, *zapB*, *zapC,* and *zapD*, genes on *zap* operon. *zapA* is responsible for controlling IgA protease expression via cell differentiation from swarmer to swimmer cells.

*P. mirabilis* has been shown to have a variety of fimbriae. The vigorous form is mannose-resistant/proteus-like (MR/P) fimbria, which is encoded by *mrpA*, *mrpB*, *mrpC*, *mrpD*, *mrpE*, *mrpF*, and *mrpG*. The *mrpA* gene contributes many virulent factors, such as bacterial adhesion, biofilm formation, and the swarming process, all of which are critical for pathogenicity [8]. *P. mirabilis* has two flagellins, *flaA* and *flaB*, which are responsible for the flagellum's whip formation (also known as *fliC1* and *fliC2*, respectively) [9]. Toxins like hemolysins, endotoxins, and proteus toxic agglutinin (Pta) are also found leading to treatment failure. *P. mirabilis* hemolytic activity is attributed to two encoded genes, *hpmA* and *hpmB*. *hpmA* gene is the main cause of tissue damage in the presence of *hpmB* [10].

Due to the increasing of the clinical significance of *P. mirabilis*, the selection of effective molecular fingerprinting methods is of significant epidemiological importance. Bacterial genotyping also opened up new avenues for epidemiological research, allowing for the discovery of clinical isolates and the assessment of their relations. There are several methods for bacterial genotyping [11]. One of the most effective techniques is PCR-based typing, which is marked by its speed, ability to type a wide range of bacterial organisms, and ease of use. Random amplified polymorphic DNA (RAPD) [12] and repetitive sequence-based PCR genomic fingerprinting (repeated sequences enterobacterial repetitive intergenic consensus sequence (ERIC), repetitive extragenic palindromic sequence (REP), and BOX A1R based repetitive extragenic palindromic PCR (BOX-PCR)) have been specifically designed for prokaryotic fingerprinting [13]. Furthermore, Ribotyping and pulsed-field gel electrophoresis (PFGE) are effective methods for characterizing *Proteus* at the species level. However, these procedures are time consuming, costly, and labor intensive, restricting their use in routine clinical laboratories. Tandem tetramer microsatellites (GACA)4, also known as intergenic single sequence repeats (ISSR), have recently provided a high level of discrimination for *P. mirabilis* [14].

The efficiency of the typing process is determined by a number of aspects, including typeability and discrimination. The proportion of bacterial isolates that can be positively typed by the typing marker is known as typeability. The number of types obtained by a typing technique and their relative frequencies can be used to quantify its discriminatory power by discrimination index (DI). Various criteria can be used to determine the effectiveness of molecular markers in determining genetic diversity as total bands, % polymorphisms, % monomorphism and index of strain diversity (ISD%) [15]. The Rand (R) and Wallace (W) coefficients can be used to determine the correspondence between typing methods quantitatively. They are used to figure out how far different typing approaches agree [16]. The Rand coefficient estimates the possibility that a pair of isolates that are typed the same by one form are both typed the same by the other [17]. The Wallace coefficient corrects the typing concordance for chance consensus by demonstrating the possibility that pairs of isolates are typed as same by separate approaches [18].

Our aim was to assess the virulence and resistance characteristics of *P. mirabilis*, as well as compare the efficacy of five PCR-based molecular methods for the characterization of clinical *P. mirabilis* isolates. Besides, an evaluation of the concordance of the used typing methods was performed.

## Materials and methods

### Bacterial isolates

During the period from August 2016 to April 2017, 300 clinical specimens were collected from Urology and Nephrology Center (UNC), Mansoura University Hospitals, Dakahlia governorate, Egypt. All isolates were checked and identified using an automated VITEK-2 system (bioMerieux, Marcy I’Etoile, France). The research proposal was approved by the research ethics committee of Faculty of pharmacy, Mansoura University (code:2021- 233).

### Determination of antimicrobial susceptibility pattern of *P. mirabilis* isolates

Susceptibility to different antimicrobials was determined by Kirby-Bauer disc diffusion technique according to the CLSI [19]. Eleven antimicrobial discs of various categories were used including; amikacin 30 µg (aminoglycosides), amoxicillin/clavulanic acid 20/10 µg (penicillin/ β-lactamase inhibitor), piperacillin/tazobactam 100/10 µg (anti pseudomonal penicillin/ β-lactamase inhibitor), ceftazidime 30 µg and cefotaxime 30 µg (extended-spectrum cephalosporins), imipenem 10 µg and ertapenem 10 µg (carbapenem), Trimethoprim/Sulfamethoxazole 1.25/ 23.75 µg (folate pathway inhibitor), ciprofloxacin 5 µg, ofloxacin 5 µg (second generation fluoroquinolone) and nitrofurantoin 300 µg (antiseptic drug); all discs were supplied from Bioanalyze^®^ products, Turkey. In this study, MDR was evaluated as resistance to at least one agent in three or more antimicrobial categories [20]. The Multiple Antibiotic Resistance Index (MARI) for each isolate was determined as follows: (the number of antibiotics against which the studied isolate is resistant divided by the total number of antibiotics evaluated) [21].

### Quantitative detection of biofilm using microtiter plate assay

Slime production and biofilm formation was assessed using the microtitre plate assay as previously described [22–24]. Using a Microtiter plate reader, the optical density of wells was determined at 540 nm (Bio Tek instruments El800, 29,274, USA). The average OD of the three wells was measured for each isolate (ODT). Three standard deviations (SD) above the mean of the negative control well are used to determine the cutoff OD (ODC). The isolates that were tested were categorized as follows: If OD_T_ ≤ OD_C_, they are non-biofilm-producer; if OD_C_ < OD_T_ ≤ 2 OD_C_, they are weakly biofilm-producer, moderately biofilm-producer if 2 OD_C_ < OD_T_ ≤ 4 OD_C_, and strongly biofilm-producer if 4 OD_C_ < ODT [25].

### Qualitative detection of virulence factors

#### Blood hemolysis

Blood hemolysis test was done by streaking each isolate on blood agar containing 5% (vol/vol) blood. Complete hemolysis (β), partial hemolysis (α) or no hemolysis (γ) was detected after incubation at 37 °C for 24 h. [1, 26].

#### Urease production

Urease production test was done by culturing each isolate on Christensen urea agar containing 40% urea supplement added to the solution (45–50 °C). The formation of a pink zone was detected after incubation at 37 °C for 24 h. [1].

#### Extracellular protease production

The protease production test was done by streaking each isolate on media containing (1% w/v) soluble casein agar as a substrate and skimmed milk powder (1% w/v). The formation of a clear zone surrounding the growth was detected indicating casein proteolysis after incubation at 37 °C for 24 h. [27, 28].

### Molecular study

#### PCR detection of virulence genes

The genomic DNA was prepared as previously reported; briefly, 5–7 fresh colonies of each isolate were picked up from nutrient agar plates, and suspended in 100 µl of sterile nuclease-free water, boiled at 95 °C for 10 min, centrifuged then the supernatant frozen at −20 °C [29].

Table 1 shows the nucleotide sequence, product size, and the annealing temperature of the primers used in this study. The PCR amplification and reaction conditions were performed as previously reported for each primer.

**Table 1:** The primer sets used for PCR amplification of 60 *P. mirabilis* isolates

Target gene	Type	Nucleotide sequence (5` to 3`)	Product size	Annealing temperature	References
Set of primers used for amplification of virulence genes					
*zapA*	F	ACCGCAGGAAAACATATAGCCC	540	59 °C	[30]
	R	GCGACTATCTTCCGCATAATCA			
*ureA*	F	GATCTGGGCGACATAATCGT	362	54.2 °C	[30]
	R	TCACCGGGGATCATGTTATT			
*ureC*	F	GTTATTCGTGATGGTATGGG	317	56.2 °C	[8]
	R	ATAAAGGTGGTTACGCCAGA			
*mrpA*	F	ACACCTGCCCATATGGAAGATACTGGTACA	550	40 °C	[8]
	R	AAGTGATGAAGCTTAGTGATGGTGATGGTGATGAGAGTAAGTCACC			
*flaA*	F	AGGATAAATGGCCACATTG	417	54.2 °C	[30]
	R	CGGCATTGTTAATCGCTTTT			
*hpmA*	F	TGGTATCGATGTTGGCGTTA	717	56.2 °C	[30]
	R	GTGGTGCCCACTTTCAGATT			
*rsbA*	F	TTGAAGGACGCGATCAGACC	467	58 °C	[8]
	R	ACTCTGCTGTCCTGTGGGTA			
Set of primers used for Typing Techniques					
RAPD	*OPZ19*	GTGCGAGCAA		40 °C	[14]
	*OPX13*	ACGGGAGCAA		40 °C	[14]
ISSR	*1*	(AC)8 T		48 to 50 °C	[14]
	*2*	(AG)8 A		48 to 50 °C	[14]
	*3*	(GA)8 T		48 to 50 °C	[14]
	*6*	(GACA)4		48 to 50 °C	[14]
ERIC	*1R*	ATGTAAGCTCCTGGGGATTCAC		48 °C	[31]
	*2*	AAGTAAGTGACTGGGGTGAGCG		48 °C	[31]
BOX	*A1R*	CTACGGCAAGGCGACGCTGACG		50 °C	[32]
REP-PCR	*1R*	IIIICGICGICATCIGGC		40 °C	[31]
	*21R*	ICGICTTATCIGGCCTAC		40 °C	[31]

#### Molecular typing

Five PCR-based typing methods RAPD, ERIC, BOX, REP, and ISSR were used to show the relatedness among isolates. The temperature profile and the reaction condition were performed as previously mentioned at Table 1 for each primer. The amplified PCR products were visualized on 1% agarose gel electrophoresis stained with ethidium bromide, and illuminated by UV transilluminator, photographed using gel documentation, and analyzed using GelJ software (V.2); similarities between isolates concerning the results of typing methods were determined by cluster analysis using distance matrix, and UPGMA software to generate dendrogram at similarity ≥ 70% [33].

#### Data analysis

For the analysis of molecular typing data, a set of variables was calculated. The typeability of each typing method was calculated as the percentage of typeable isolates among the total isolates. The index of strain diversity (ISD) was assessed according to the following equation: ISD [%] = [number of genotypes ÷ total number of isolates]*100% [34]**.** The discriminatory index (DI) was calculated from the relative frequencies of the different profiles obtained by a given method using Simpson's diversity index (SID), and Shannon’s Index of Diversity (H) where they were calculated using software described by [35].

The congruence assessments between typing methods were performed using the Rand’s coefficient (R), and for advanced comparison the Wallace (W) coefficients were also performed using the software described by [35].

### Statistical analysis

To collect descriptive results, data were tabulated, coded, and evaluated using the computer software SPSS version 26.0. The following descriptive statistics were calculated: Interquartile distribution, median (IQR), and Recurrence (Number/percent).

Analytical statistics: The magnitude of variance was checked using one of the following tests in the statistical comparison between the various groups: Mann Whitney, Pearson's chi-square test (X^2^-value) or Fisher exact, and Monte-Carlo. Spearman’s correlation coefficient test was used to correlate different parameters. A P value < 0.05 was considered statistically significant.

## Results

### Collection, isolation, and identification of clinical isolates

Three hundred clinical specimens were collected from different clinical cases of urinary tract infections at Urology and Nephrology Center, Mansoura University from August 2016 to April 2017. Sixty clinical isolates of* P. mirabilis* were identified. Isolates were obtained from patients suffering from urinary tract infections. Supplementary Table 1 illustrated the isolates, the gender, age, and the clinical case of the patient with the date of isolation.

The most prevalent diagnosis of included patients was renal stone cases which represented 16.70% followed by hypospadias cases (13.30%), then post- repair of vesicocutaneous fistula (8.33%). The majority of cases are represented by males (72%). The cases were classified into three groups according to age; the 1st group included most of the cases < 18 years (40%), the 2nd group included cases from 18 to 60 years old (35%), and the 3rd group included cases ˃ 60 years old (25%).

### Determination of antimicrobial susceptibility pattern of all *P. mirabilis* isolates:

The antimicrobial susceptibility patterns of *P. mirabilis* isolates were evaluated by the disc-diffusion method. The highest resistance level was exhibited by nitrofurantoin (98.3%) followed by trimethoprim/sulfamethoxazole (81.7%). Moderate levels of resistance to ofloxacin, ciprofloxacin, amoxicillin/ clavulanic acid, cefotaxime, and ceftazidime (60.0%, 58.3%, 45.0%, 41.7%, and 40.0% respectively) were recorded. A low level of resistance was observed with amikacin, imipenem, piperacillin/ tazobactam and ertapenem (8.3%, 11.7%, 11.7%, and 13.3% respectively).

Table 2 shows the antibiotic resistance pattern of the tested isolates. The isolates showed 22 antibiotypes (A). The most predominant antibiotype among isolates was A13 (16.67%) with multiple antibiotics resistance index (MAR) of 0.36, followed by A5 with a prevalence 15%, and MAR index = 0.64. MAR index ≥ 0.5 was found in A1-A10 (40% of isolates). In addition, 38.3% of isolates were distributed in A11-A20 with MAR > 0.2.

**Table 2:**
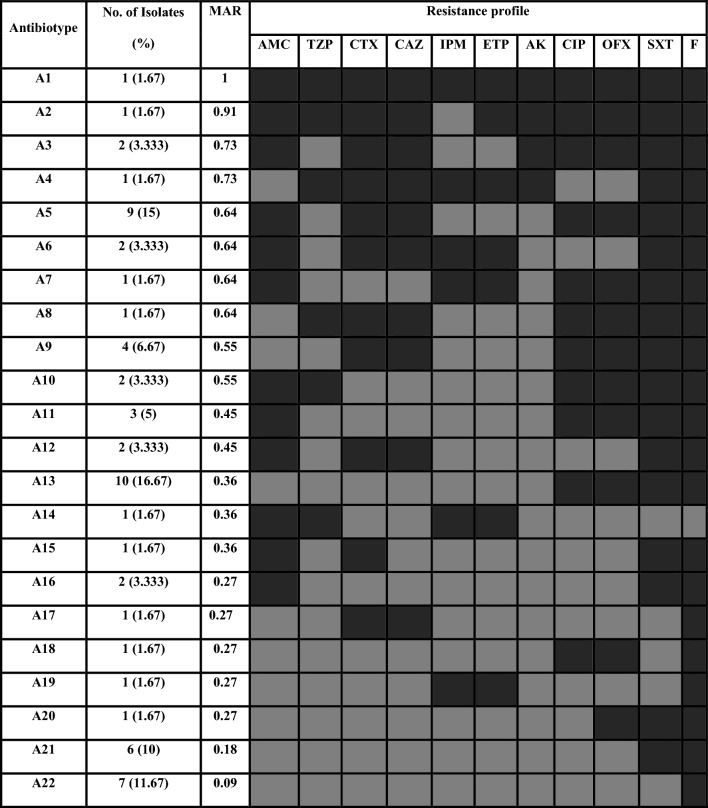
Distribution of antibiotype patterns among *Proteus mirabilis*

*AMC*: Amoxicillin/ clavulanic Acid, *TZP*: pipracillin/ tazobactam, *SXT*: Trimethoprim/ sulfamethoxazole, *CTX*: Cefotaxime, *CIP*: Ciprofloxacin, *AK*: Amikacin, *CAZ*: Ceftazidime, *ETP*: Etrapenem, *IPM*: Imipenem, *OFX*: ofloxacin, *F*: Nitrofurantoin. *MAR index*: Multiple antibiotic resistance index: (Grey filled Square)Sensitive, (Black filled Square )Resistance

Moreover, 44 (73.33%) isolates were MDR; distributed with the highest percentage 38.60% in the median age 2nd group (gp), followed by the youngest age (1st gp) representing 36.40%, and 25.00% in the elder age (3rd gp). In addition, it was found that cefotaxime, ciprofloxacin, ofloxacin, and amoxicillin/ clavulanic acid were significantly related to MDR with *P*-value < 0.001. Also, ceftazidime, and trimethoprim/ sulfamethoxazole were significantly associated with MDR (*P*-value = 0.001).

**Fig. 1:**
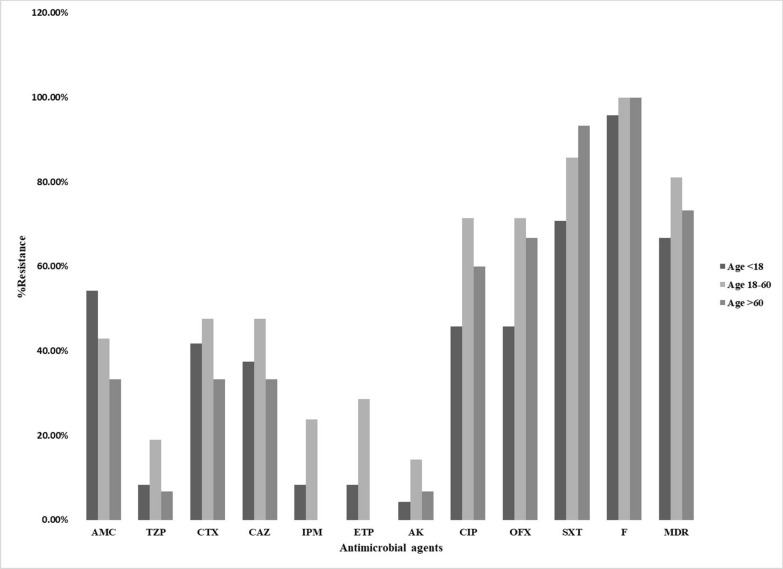
Distribution of tested antibiotics according to the age classification

The distribution of tested antibiotics according to the age classes is illustrated in Fig. 1. The children 1st gp showed high resistance to nitrofurantoin, trimethoprim/ sulfamethoxazole, and amoxicillin/ clavulanic acid (95.8%, 70.8%, and 54.2% respectively) and low resistance to amikacin (4.2%). The median age 2nd gp showed high resistance to nitrofurantoin, trimethoprim/ sulfamethoxazole, ofloxacin, and ciprofloxacin (100%, 85.7%, 71.4% and 71.4% respectively) and low resistance to amikacin (14%). Finally; the elder age 3rd gp showed high resistance to nitrofurantoin, trimethoprim/ sulfamethoxazole, ofloxacin, and ciprofloxacin (100%, 93.3%, 66.7% and 60%) and no resistance to imipenem, and ertapenem (0.00%). In addition, Imipenem and ertapenem were significantly related to the elder age 3rd gp with *P*-value = 0.017* and < 0.001* respectively.

### Detection of biofilm and virulence factors

Biofilm formation of all isolates was tested using the microtiter plate assay; 21/60 (35%) of isolates were strong biofilm-producer, 19 (31.67%) were moderate biofilm-producer, 18 (30%) were weak biofilm-producer and 2 (3.333%) were non-biofilm-producer. All isolates were positive urease, protease, and hemolysin producers. All isolates produced β hemolysins.

**Fig. 2:**
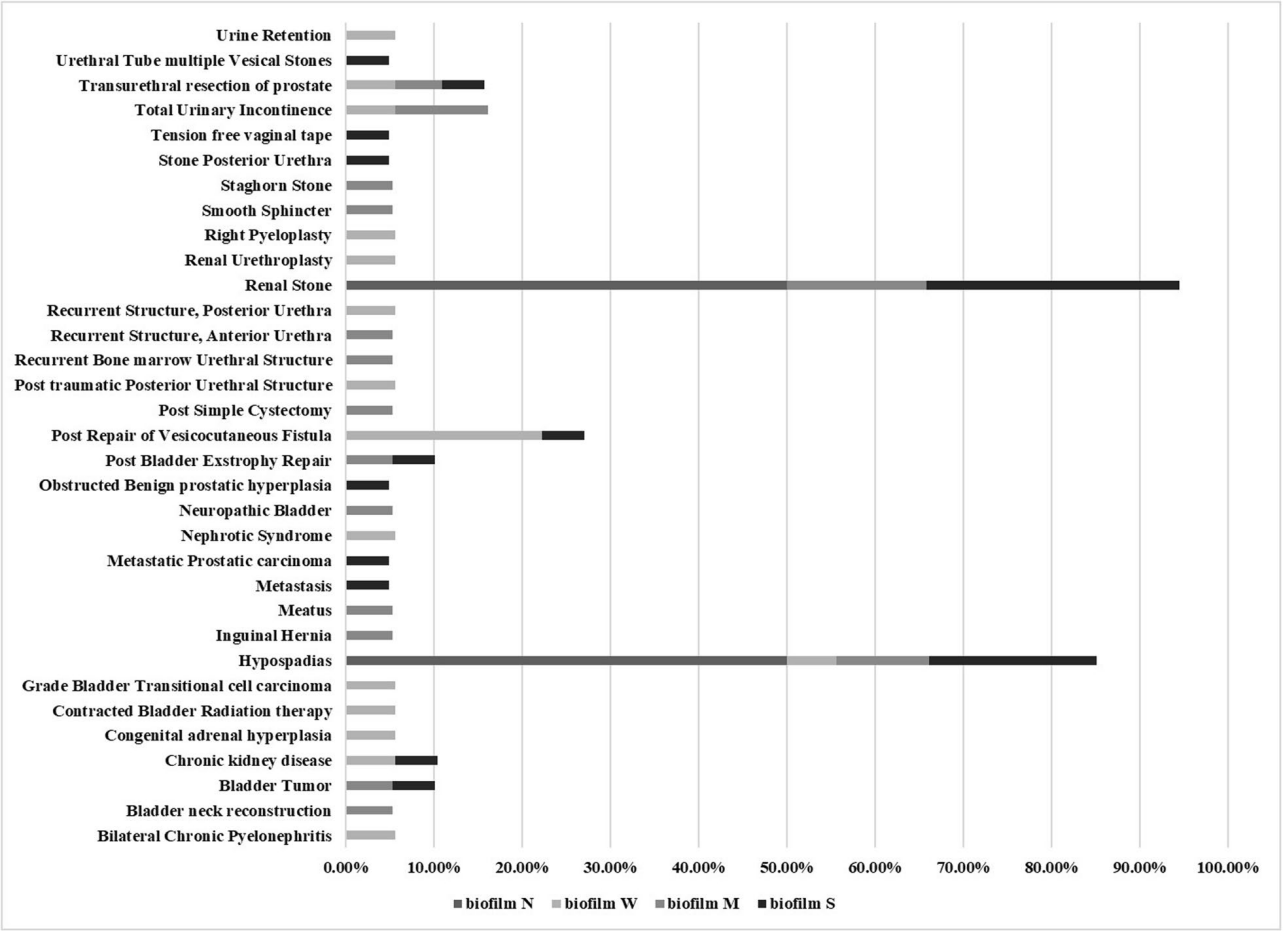
Relation between clinical cases and type of biofilm-producer

**Fig. 3:**
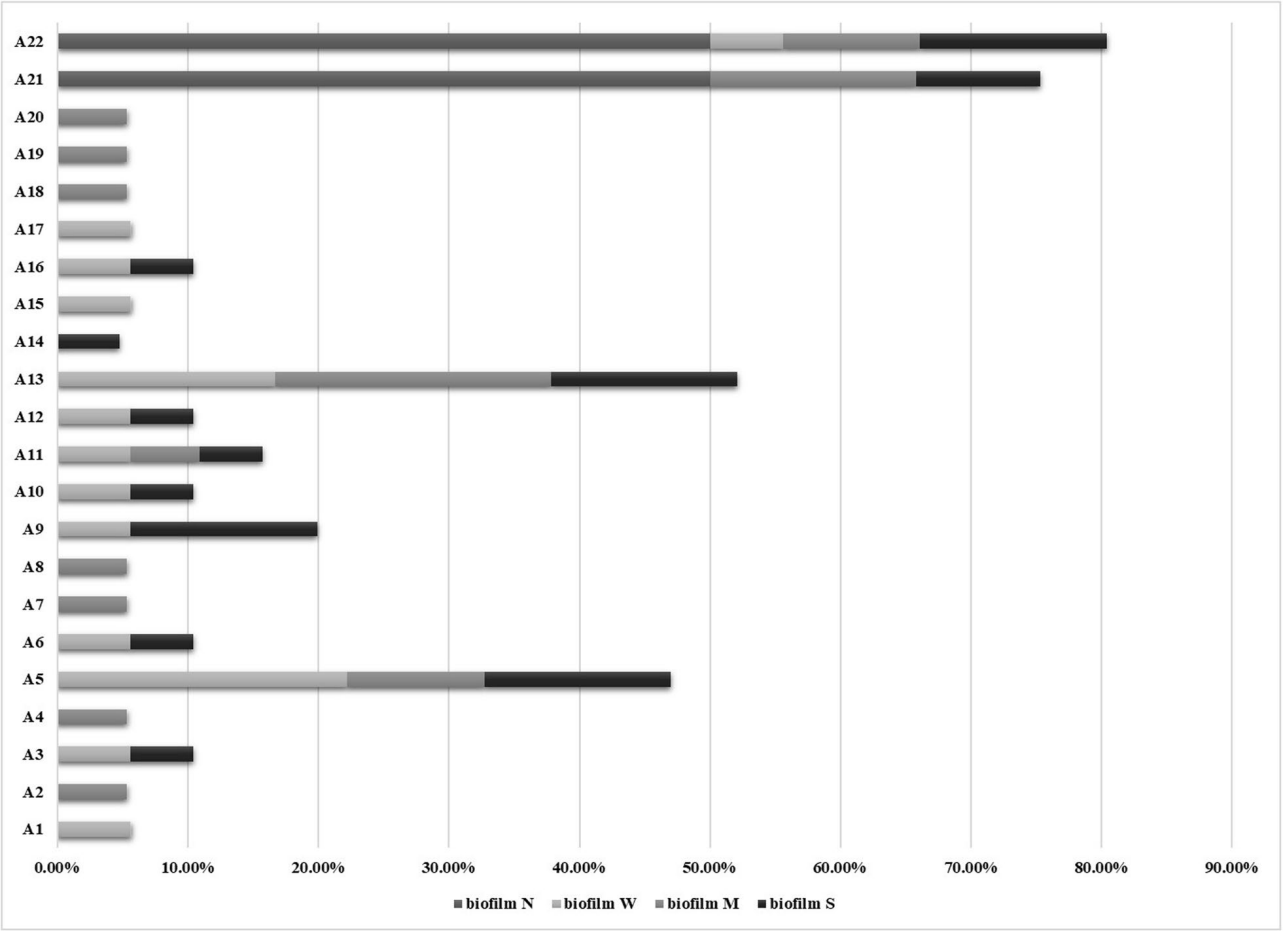
Relation between antibiotype and type of biofilm-producer

Most abundant clinical cases (bladder tumor, hypospadias, metastasis, renal stone….) were from moderate to strong biofilm-producer illustrated in Fig. 2. Also; antibiotypes patterns A5, A9, A13, A21, and A22 showed high percent for moderate to strong biofilm-producer and all non- biofilm-producers were in A21 and A22 represented in Fig. 3.

We found that biofilm was associated significantly with MDR (*P*-value = 0.017); where 36.4% of either strong and weak biofilm-producers, and 27.3% of moderate biofilm-producers were MDR while non-biofilm-producers were non-multi drug resistance (NMDR).

### PCR detection of virulence genes

**Fig. 4:**
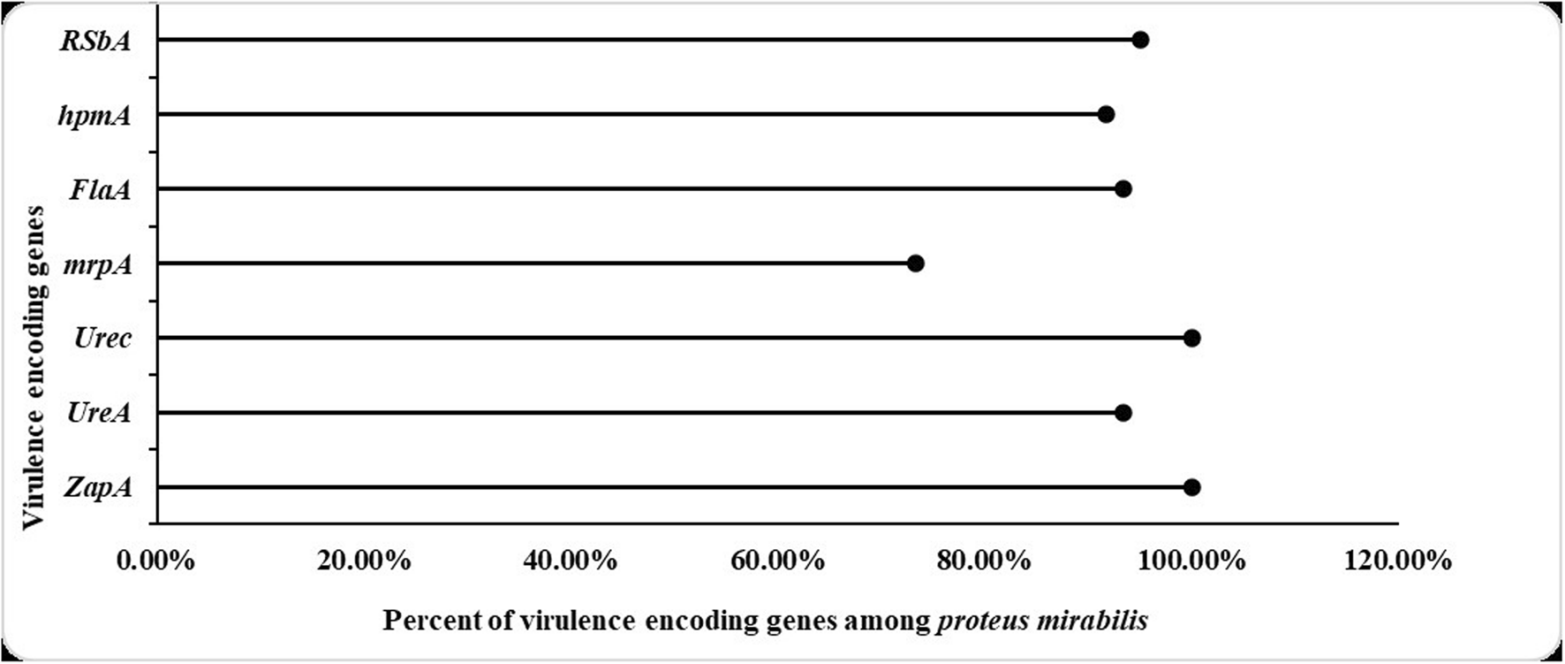
Lollipop plot of the prevalence of each virulence gene in all isolates

Different virulence genes (*zapA, ureA, ureC, mrpA, flaA, hpmA, rsbA*) were detected by PCR. It was found that *zapA* and *ureC* recorded the highest percentage of the tested virulence genes as they were harbored by all isolates, followed by *rsbA* (95%), *ureA* & *flaA* (93%), *hpmA* (91.7%) and *mrpA* (73.3%) as shown in Fig. 4.

*P. mirabilis* isolates were classified into nine different virulence gene patterns (V). The most prominent pattern was V1 represented by 38 (63.33%) isolates that harbored all tested virulence gene; distributed into 14 out of 24 isolates (58.3%) in the youngest age (1st gp), 13 out of 21 isolates (61.90%) in the median age (2nd gp) and the highest percent in the elder age (3rd gp) about 11 out of 15 isolates (73.3%). The second prominent pattern was V2 it was found in 13 (21.67%) isolates; all of them harbored all tested genes except *mrpA*, and according to age, five isolates (20.8%) were in the 1st gp, six isolates (28.6%) were in the 2nd gp, and the least value two isolates (13.3%) were in the 3rd gp. Pattern (V3 and V4) each represented in two (3.33%) isolates distributed among 1st and 2nd gp, V5, V6, V7, V8 and V9 each showed different pattern representing in only one (1.67%) isolate separately shown in Table 3.

**Table 3:** Distribution of virulence patterns among *P. mirabilis* isolates according to the classification groups of age:

Pattern	Gene profile	No. of isolates	Age groups		
			1st No (%)	2^nd^ No (%)	3^rd^ No (%)
V1	All genes	38	14 (58.3%)	13 (61.9%)	11 (73.3%)
V2	*zapA, ureA, ureC, flaA, hpmA, rsbA*	13	5 (20.8%)	6 (28.6%)	2 (13.3%)
V3	*zapA, ureC, mrpA, flaA, hpmA, rsbA*	2	1 (4.2%)	1 (4.8%)	0 (0.00%)
V4	*zapA, ureA, ureC, mrpA, flaA, rsbA*	2	1 (4.2%)	1 (4.8%)	0 (0.00%)
V5	*zapA, ureC, mrpA, flaA, hpmA*	1	0 (0.00%)	0 (0.00%)	1 (6.7%)
V6	*zapA, ureA, ureC, mrpA, rsbA*	1	1 (4.2%)	0 (0.00%)	0 (0.00%)
V7	*zapA, ureA, ureC, hpmA, rsbA*	1	0 (0.00%)	0 (0.00%)	1 (6.7%)
V8	*zapA, ureA, ureC*	1	1 (4.2%)	0 (0.00%)	0 (0.00%)
V9	*zapA, ureC*	1	1 (4.2%)	0 (0.00%)	0 (0.00%)

### Molecular typing and data analysis

Eleven typing primers of five PCR-based typing methods were used. They produced multiple band profiles with a variable number and molecular weights of amplified DNA fragments.

According to typeability, ERIC2 and REP-PCR21R recorded the highest values (98.33%), followed by ERIC IR and ISSR2 (90.00%) and REP-PCR1R (88.33%). The lowest typeability was recorded with RAPD primers (48.33 and 46.67% for OPX13 and OPZ19, respectively). When comparing the used primers according to ISD%, ERIC 2 and ERIC IR record the highest values (95% and 85%, respectively). ISSR 3 and REP-PCR21R recorded the lowest ISD% (23.33% and 25%, respectively) as illustrated in Table 4.

**Table 4:** Typing methods, typeability, ISD% and clusters at different cutoff of the dendrogram

Typing method		Typeability (%)	ISD %	No of clusters at 70%	No of clusters at 80%	No of clusters at 90%	No of clusters at 100%
*RAPD*	*OPZ19*	46.67	46.67	28	28	28	0
	*OPX13*	48.33	43.33	20	25	26	2
*ISSR*	*1*	85	53.33	23	33	33	9
	*2*	90.00	68.33	14	25	38	10
	*3*	55.00	23.33	9	14	14	7
	*6*	73.33	60	21	32	36	6
*ERIC*	*1R*	90.00	85	33	48	51	2
	*2*	98.33	95	24	46	57	2
*BOX- A1R*		70.00	63.33	21	32	38	3
*REP-PCR*	*1R*	88.33	81.67	27	41	46	3
	*21R*	98.33	25	7	22	24	14

ISD: Index of strain diversity

**Fig. 5:**
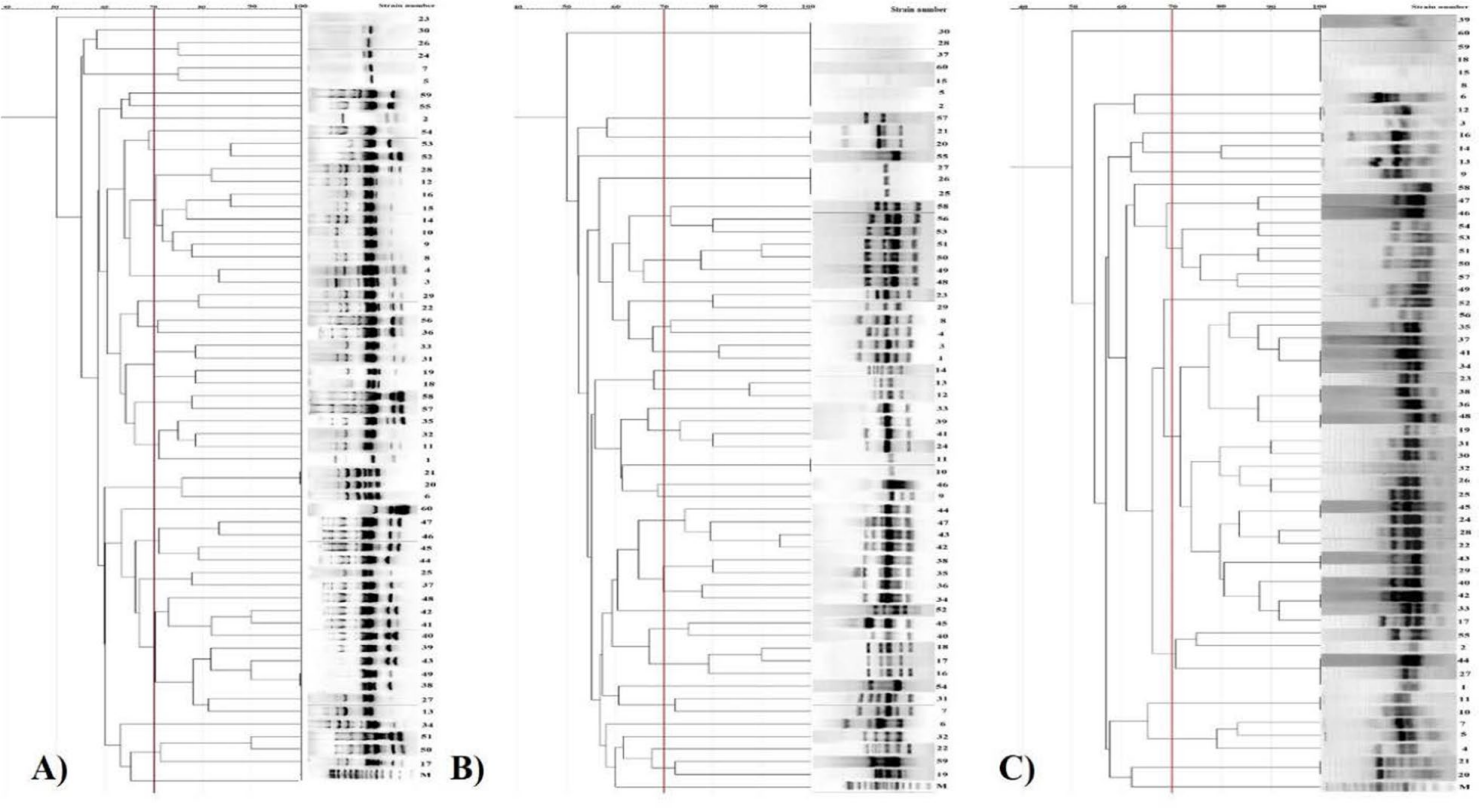
Dendrograms of *P. mirabilis* isolates were generated by Gel-J software with arithmetic mean using Jaccard coefficients (band tolerance 1.0%; optimization1.0; cut-off value 70%)** A**. *ERIC2-PCR*,** B**. *REP-PCR 1R* and** C**. *ISSR2*

Dendrograms of the *P. mirabilis* isolates were performed for 11 selected typing primers at different cutoff (70%, 80%, 90% and 100%) to compare the different genetic fingerprinting profiles obtained by different typing techniques (Fig. 5). A total of 28 clusters were identified and grouped with *RAPD OPZ19* at cutoff 70%, 80% and 90% showing high similarity between them and when we increased the cutoff to 100%, no clusters obtained. In *RAPD OPX13* upon increasing the percent of cutoff (70%, 80%, and 90%) respectively; the number of clusters increased (20, 25 and 26 clusters) respectively. Finally; two similar groups only at 100% cutoff. All the primers represented the lowest number of clusters at cutoff 70% and the highest at 90% cutoff. The number of clusters is directly proportional by increasing the percentage of cut-off. These results are briefly shown at Table 4. Isolates no. 20 and 21 show 100% similarity by three different typing methods as shown in (Fig. 5). Finally; By calculating clusters at 100% cut-off on dendrograms; the most prominent primers showing similarity was *REP-PCR 21R* representing 14 clusters followed by *ISSR2* representing 10 clusters then by *ISSR 1*, *ISSR3* and *ISSR6* (9, 7 and 6 clusters respectively). Both *BOX- A1R* and *REP-PCR 1R* have the same number of clusters (3), and both *ERIC1R* and *ERIC 2* have two clusters only. Finally; RAPD (OPZ19) have no clusters at 100% cut-off. Hence, different cut-off % facilitate the comparison of different typing techniques to obtain the most discriminatory power and reproducibility.

Different polymorphic bands (PB) and monomorphic bands (MB) were calculated across RAPD, ISSR, ERIC, BOX-AIR and REP-PCR and represented in Table 5. ERIC primers showed the highest PB% (77.77% and 66.67%) with the highest effective multiplex ratio (EMR) values (5.4 and 5.3) for *ERIC 1* and *ERIC 2* respectively**.** RAPD primers gave the highest MB% (66.67%) and the lowest PB% (33.33) with EMR (1 and 0.7 for OPZ19 and OPX13, respectively) showing less reproducible and discriminatory power. The lowest EMR was detected for ISSR 3 as it was 0.5 (Table 5).

**Table 5:** Polymorphism, monomorphism and effective multiplex ratio of the used PCR-based typing methods

Typing method		TB	MB	PB	MB%	PB%	EMR
*RAPD*	*OPZ19*	9	6	3	66.67	33.33	1
	*OPX13*	6	4	2	66.67	33.33	0.7
*ISSR*	*1*	5	3	2	60	40	0.8
	*2*	6	3	3	50	50	1.5
	*3*	2	1	1	50	50	0.5
	*6*	5	2	3	40	60	1.8
*ERIC*	*1R*	9	2	7	22.22	77.77	5.4
	*2*	12	4	8	33.33	66.67	5.3
*BOX- A1R*		9	4	5	44.44	55.56	2.8
*REP-PCR*	*1R*	7	3	4	42.86	57.14	2.3
	*21R*	5	2	3	40	60	1.8

*TB*: Total bands, *MB*: Monomorphic bands, *PB*: Polymorphic bands, *PM%*: percentage of monomorphic bands, *PB%*: percentage of polymorphic bands, *EMR*, effective multiplex ratio

Simpson's Index of Diversity (SID) and Shannon's Index of Diversity (H) were calculated for all the used primers where the highest SID were for ERIC typing method (SID = 0.999 and 0.989 for *ERIC 2* and *ERIC 1R*, respectively), *REP-PCR 1R* (SID = 0.985), *ISSR* (SID = 0.983, o.955 and 0.934 for *ISSR2*, *ISSR1* and ISSR6) and *BOX-AIR* (SID = 0.911%) with 95% confidence level (Table 6). The discriminatory power calculated by H was similar to results obtained by SID as shown in Table 6.

**Table 6:** Discriminatory power of PCR-based typing method calculated with Simpson's Index of Diversity and Shannon’s Index of Diversity

Typing method		No. of patterns	Simpson’s ID	C (95%)	(H)
*RAPD*	*OPZ19*	49	0.72	(0.587–0.853)	0.667
	*OPX13*	15	0.735	(0.607–0.863)	0.68
*ISSR*	*1*	38	0.955	(0.929–0.980)	0.904
	*2*	57	0.983	(0.970–0.996)	0.962
	*3*	51	0.776	(0.675–0.876)	0.737
	*6*	36	0.934	(0.883–0.985)	0.882
*ERIC*	*1R*	14	0.989	(0.976–1.000)	0.971
	*2*	41	0.999	(0.997–1.000)	0.997
*BOX- A1R*		32	0.911	(0.844–0.977)	0.853
*REP-PCR*	*1R*	26	0.985	(0.969–1.000)	0.963
	*21R*	28	0.84	(0.763–0.916)	0.802

Simpson’s index of diversity: SID, CI; 95% confidence interval, H: Shannon index of diversity

### Concordance between the different typing methods using Rand and Wallace coefficients’

To measure the congruence between the patterns achieved by different typing methods, Rand (R) and Wallace (W) were calculated. The values of R obtained indicated the highest overall congruence was determined for R *ERIC1R-ERIC2*, followed by R *ERIC2*- *REP-PCR1R*, R *ISSR2- ERIC2*, R *ERIC1R- REP-PCR1R*, and R *ISSR2- ERIC1R*; 0.988, 0.985, 0.983, 0.975 and *0*.973, respectively above the recommended value (0.95) (Table 7).

**Table 7:** The congruence among different typing methods according to the Rand coefficient

	*RAPD OPX13*	*RAPD OPZ19*	*ISSR 1*	*ISSR 2*	*ISSR 3*	*ISSR 6*	*ERIC1R*	*ERIC 2*	*BOX- A1R*	*REP-PCR 1R*
*RAPD OPX13*										
*RAPD OPZ19*	0.716									
*ISSR 1*	0.727	0.708								
*ISSR 2*	0.733	0.711	0.938							
*ISSR 3*	0.649	0.682	0.75	0.767						
*ISSR 6*	0.697	0.721	0.895	0.919	0.765					
*ERIC1R*	0.729	0.715	0.944	**0.973**	0.772	0.929				
*ERIC 2*	0.734	0.72	**0.954**	**0.983**	0.777	**0.934**	**0.988**			
*BOX- A1R*	0.68	0.695	0.873	0.897	0.724	0.869	0.905	0.91		
*REP-PCR 1R*	0.727	0.708	0.941	**0.972**	0.768	0.92	**0.975**	**0.985**	0.898	
REP-PCR 21R	0.65	0.666	0.806	0.832	0.699	0.804	0.841	0.84	0.784	0.828

Bold indicates higher and more accurate values

The results obtained for W for the comparison of the different typing methods are shown in Table 8. The values of concordance between the type assignment done by *ERIC2* and *ISSR3* as defined by W are relatively high (W *ISSR3- ERIC2* (1.000–1.000) 1). REP-PCR21R can be predicted by ERIC1R (W = 0.579). Moreover, OPZ19, ISSR2, ISSR6, REP-PCR1R and REP-PCR21R can be predicted by ERIC2 with W = 0.5. The values of R and W between the different typing methods and the patterning performed by *ERIC* and *REP-PCR* typing methods were generally higher and more accurate than comparing the data visually.

**Table 8:** The congruence between different typing methods using the Wallace coefficient

Typing methods	Wallace coefficient (95%)										
	RAPD		ISSR				ERIC		BOX- A1R	REP-PCR	
	OPX13	OPZ19	1	2	3	6	1R	2		1R	21R
RAPD OPX13		0.493	0.07	0.028	0.26	0.053	0.009	0	0.064	0.013	0.143
		(0.272–0.713)	(0.000–0.142)	(0.000–0.067)	(0.115–0.405)	(0.000–0.114)	(0.000–0.032)	(0.000–0.000)	(0.000–0.138)	(0.000–0.043)	(0.039–0.247)
RAPD OPZ19	0.466		0.06	0.014	0.333	0.121	0.01	0.002	0.115	0.006	0.19
	(0.249–0.682)		(0.000–0.130)	(0.000–0.038)	(0.161–0.504)	(0.017–0.225)	(0.000–0.033)	(0.000–0.019)	(0.009–0.221)	(0.000–0.027)	(0.071–0.308)
ISSR 1	0.412	0.375		0	0.212	0.075	0	0	0.087	0.013	0.125
	(0.242–0.583)	(0.209–0.541)		(0.000–0.000)	(0.080–0.345)	(0.000–0.187)	(0.000–0.000)	(0.000–0.000)	(0.000–0.211)	(0.000–0.097)	(0.000–0.258)
ISSR 2	0.433	0.233	0		0.233	0.067	0.033	0.033	0.1	0.1	0.267
	(0.186–0.680)	(0.031–0.436)	(0.000–0.000)		(0.042–0.425)	(0.000–0.218)	(0.000–0.188)	(0.000–0.188)	(0.000–0.269)	(0.000–0.269)	(0.138–0.396)
ISSR 3	0.307	0.416	0.043	0.018		0.123	0.015	0.005	0.083	0.015	0.186
	(0.128–0.487)	(0.211–0.621)	(0.003–0.083)	(0.000–0.048)		(0.010–0.236)	(0.000–0.052)	(0.000–0.030)	(0.000–0.170)	(0.000–0.045)	(0.067–0.306)
ISSR 6	0.214	0.513	0.051	0.017	0.419		0.043	0.009	0.188	0.009	0.231
	(0.023–0.404)	(0.234–0.792)	(0.000–0.127)	(0.000–0.079)	(0.170–0.667)		(0.000–0.103)	(0.000–0.066)	(0.025–0.351)	(0.000–0.066)	(0.041–0.421)
ERIC1R	0.211	0.263	0	0.053	0.316	0.263		0	0.211	0	**0.579**
	(0.000–0.496)	(0.000–0.552)	(0.000–0.000)	(0.000–0.270)	(0.000–0.670)	(0.071–0.455)		(0.000–0.000)	(0.011–0.410)	(0.000–0.000)	(0.221–0.937)
ERIC 2	0	**0.5**	0	**0.5**	**1**	**0.5**	0		0	**0.5**	**0.5**
	(0.000–0.000)	(0.000–1.000)	(0.000–0.000)	(0.000–1.000)	(1.000–1.000)	(0.000–1.000)	(0.000–0.000)		(0.000–0.000)	(0.000–1.000)	(0.000–1.000)
BOX- A1R	0.19	0.361	0.044	0.019	0.209	0.139	0.025	0		0.013	0.19
	(0.010–0.370)	(0.109–0.613)	(0.000–0.122)	(0.000–0.068)	(0.039–0.379)	(0.018–0.261)	(0.000–0.084)	(0.000–0.000)		(0.000–0.059)	(0.055–0.324)
REP-PCR 1R	0.231	0.115	0.038	0.115	0.231	0.038	0	0.038	0.077		0.115
	(0.000–0.535)	(0.000–0.352)	(0.000–0.214)	(0.000–0.282)	(0.006–0.456)	(0.000–0.214)	(0.000–0.000)	(0.000–0.182)	(0.000–0.256)		(0.000–0.290)
REP-PCR 21R	0.236	0.331	0.035	0.028	0.261	0.095	0.039	0.004	0.106	0.011	
	(0.074–0.398)	(0.140–0.522)	(0.000–0.086)	(0.000–0.070)	(0.117–0.404)	(0.001–0.189)	(0.000–0.102)	(0.000–0.036)	(0.007–0.205)	(0.000–0.045)	

Bold indicates higher and more accurate values

## Discussion

*Proteus mirabilis* is a Gram-negative bacterium that is notorious for its ability to swarm firmly across surfaces in a striking bull’s eye pattern. Clinically, it exhibits an extraordinary lifestyle as uropathogen invading human urinary tracts. *P. mirabilis* was the 2nd most predominant uropathogen (5.2%) in the urine of male patients using indwelling catheters [36]. It is considered a model microorganism for urease-producing pathogens forming crystalline biofilms on indwelling urinary catheters, often leading to poly-microbial infection. Recent studies have illustrated how *P. mirabilis* causes all of these diseases. Especially, the discovery that this bacterium forms huge clusters in the bladder lumen forming stones, bladder tumors and several diseases mentioned some of them in our study. These clusters, and other steps of infection, require several virulence factors like urease, MR/P fimbriae, and hemolysins. To the best of our knowledge, this is the first study that investigates the virulence factors, and antimicrobial resistance and tries to evaluate the different molecular typing methods in the discrimination of *P. mirabilis*. In this study, 60 *P. mirabilis* were identified with incidence of 20%. In this study; The most prevalent diagnosis of isolated samples is Renal Stone cases as it represented 16.70% followed by Hypospadias cases (13.30%). In [37] study; 50% percent of urolithiasis cases are recurrent illnesses, leading to the loss of kidney if not properly treated. Several uropathogens are associated with the formation of bacteria-induced urinary stones but *P. mirabilis* is the most commonly caused (70%) among the tested uropathogens; as only *P. mirabilis* produced crystallization within the epithelial cells due to the increased number of intracellular bacteria and their urease activity. In Hungary; a study by [38], proved that the correlation between the existence of significant risk factors, and underlying illnesses (age, catheterization, and inpatient/outpatient status) with *Proteus* urinary tract infections could not be evaluated. The high-rate level of bacteriuria in elderly patients is considered an important role during isolation of *Proteus*.

Our isolates showed high resistance to nitrofurantoin (98.3%) and trimethoprim/ sulfamethoxazole (81.7%). They show moderate levels of resistance to ofloxacin, ciprofloxacin, amoxicillin/ clavulanic acid, cefotaxime and ceftazidime (60.0%, 58.3%, 45.0%, 41.7% and 40.0% respectively). A Low level of resistance was observed with amikacin, imipenem, piperacillin/ tazobactam and ertapenem (8.3%, 11.7%, 11.7% and 13.3% respectively). Finally; 73.33% of isolates were MDR. There is a statistically significant relation between the age of patients (15–65 years) and resistance to ofloxacin (*P*-value = 0.024). it was found that cefotaxime, ciprofloxacin, ofloxacin and amoxicillin/ clavulanic acid were significantly related to MDR with *P*-value < 0.001. Also, ceftazidime and trimethoprim/ sulfamethoxazole were significantly associated with MDR (*P*-value = 0.001) [39]. Reported similar results where 55.6% were MDR. MDR *P. mirabilis* isolates were associated significantly (P < 0.001) with resistance to penicillins (amoxicillin/clavulanic acid and piperacillin), cephalosporins (cefuroxime, ceftriaxone and ceftazidime), gentamicin, ciprofloxacin and trimethoprim/ sulfamethoxazole. These results are in agreement with the results obtained by [40]; showing that the highest resistance rate was found to trimethoprim/ sulfamethoxazole combination. A study performed by [36] showed variable resistance rates for ampicillin and amoxicillin (40%), ticarcillin (42%), gentamicin (18%), nalidixic acid (26%), norfloxacin (22%), ciprofloxacin (21%), trimethoprim/ sulfamethoxazole (32%), and fosfomycin (19%) [36].

In our study; the antibiotic resistance patterns of the tested isolates were evaluated. The most prominent antibiotype was A13 representing 16.67% of isolates with MAR index = 0.36. Followed by A5 antibiotype pattern (15%) with MAR index = 0.64. In addition, several antibiotype (A1-A10) had MAR index > 0.5. Calculation of MAR index is useful for a proper treatment of UTI patients and limits the use of resistant antibiotics so preventing furthermore development of bacterial drug resistance. Similar results were reported by [21]. The MAR index is a good risk assessment tool and the value of the MAR index = 0.2 has been applied to differentiate low and highly risk regions where antibiotics are overused [41, 42]. This analysis gives a good idea of the number of bacteria showing antibiotic resistance in the zone of the exposure study.

The distribution of tested antibiotics according to the age classes was studied. The elder age (3rd gp) comprised the majority of isolates resistant to nitrofurantoin and trimethoprim/ sulfamethoxazole. Moreover, it was associated with absence of resistance to imipenem and ertapenem (*P*-value = 0.017 and < 0.001 respectively). The median age (2nd gp) contained most of the isolates resistant to ofloxacin, ciprofloxacin, cefotaxime, ceftazidime, amikacin, piperacillin/tazobactam, imipenem and ertapenem as compared to the other two groups. The children (1st gp) contained isolates resistant to all used antimicrobials but to a lesser extent than the 2nd and 3rd groups except for amoxicillin/ clavulanic acid. [43] had reported similar results that the distribution of antimicrobial resistance between age groups was not statistically significant.

In our study; biofilm formation of isolates was achieved in 96.67% except 2 isolates (3.33%) (non-biofilm-producer). Twenty-one (35%) isolates were strong biofilm-producer, 19 (31.67%) isolates were moderate biofilm-producer and 18 (30%) isolates were weak biofilm-producer. That may be attributed to the fast growth of *P. mirabilis* that provides a high yield of biofilm that is vital in the host invasion [44]. [43] stated that all tested *P. mirabilis* isolates were biofilm-producers with 71.8% strong, 21.8% moderate and 6.4% weak biofilm-producers. [40] showed that isolates formed biofilms with very strong intensity, strong intensity, and moderate intensity (73.2%, 25.7% and 1.1% respectively. We found that biofilm was associated significantly with MDR (*P*-value = 0.017); where 36.4% of either strong and weak biofilm-producers and 27.3% of moderate biofilm-producers were MDR while non-biofilm-producers were NMDR. [44] reported that stronger biofilm-producers were also MDR *P. mirabilis*.

In this study, all of the tested isolates produced urease, protease and β hemolysins. Similar results were reported by [43]. Weak biofilm-producers were mainly in age groups 1–40 years old [43]. Similar to our results, [45] showed that all *P. mirabilis* isolates generated strong production of urease and β-hemolytic action. Al- Mayahi showed that 89.5% were hemolysin producers and 100% were positive urease producers [1].

Regarding the detection of virulence genes by PCR, *zapA* and *ureC* recorded the highest percentage of the tested virulence genes as they were harbored by all isolates (100%), followed by *rsbA* (95%), *ureA* & *flaA* (93%), *hpmA* (91.7%) and *mrpA* (73.3%). It was found that *hpmA* was significantly related to strong biofilm-producers with *P*-value < 0.001. the mannose-resistant *Proteus*-like adhesion (*mrpA*), *P. mirabilis* fimbriae (*pmfA*) and uroepithelial cell adhesion (*uca* play a crucial role in the catheter-associated biofilm formation, and the bladder and kidney colonization, respectively. *zapA, hpmA, hpmB* and *ureC* are engaged in the immune system evasion and/or iron acquisition, with *zapA* also involved in the swarmer cell differentiation and swarming behavior [44]. Sun et al. 2020 study showed similar results as biofilm formation was significantly related to the expression of *atfA*, *ureC, pmfA*, *zapA*, *rsmA*, *mrpA*, and *hmpA* with *P*-value < 0.05 [46].In addition, it reported that 92.05% of the *P. mirabilis* isolates were biofilm-producers and 38.27%, 48.15%, 13.58%, and 7.95% were strong, moderate, weak and non-biofilm-producers respectively. A study conducted in Brazil showed that all isolates were positive for (*atf*A, *ptA, pmfA, ireA, zapA, mrpA, and hpmA*) virulence genes [40]. Ali and Yousef, 2015 reported that *zapA, hpmA* and *ureC* were represented by 100% of isolates while *ureA* and *flaA* were found in 96.66% and 86.66% of isolates, respectively [30]. Another study showed that *mrpA* and *rsbA* were found in 35%, and 53% of isolates; respectively [47].

All *P. mirabilis* isolates were distributed into nine different virulence gene patterns. The most abundant patterns were V1 and V2 in 38 and 13 isolates respectively.

Molecular epidemiology stresses the role of environmental and genetic aspects that affect at molecular levels the disease process. It results in the determination of disease etiology, penetrance and distribution pattern in families and population. It investigates the molecular mechanisms and specific genes involved in disease risk, which aids in understanding disease pathogenesis. Molecular epidemiology gives a new way to better explain the disease processes and the recommendations needed for disease prevention and care. Molecular epidemiology should not be regarded as just another method or tool, but as an established control in epidemiology that can progress our knowledge about these diseases in ways that were impossible in the past [48].

Various techniques have been used in molecular epidemiology such as restriction endonucleases of genomic and plasmid DNA, southern hybridization, pulsed gel electrophoresis (PFGE), PCR-based approaches for plasmid and chromosomal profiling, microarray, and the most recent revolution, whole genome sequencing. In our study various molecular PCR-based typing techniques, such as RAPD, ISSR, ERIC-PCR, BOX-PCR and rep-PCR for *P. mirabilis* have been developed used. Various approaches have been utilized to characterize *Proteus mirabilis*. An important aspect is how a single method or a combination of methods can offer insights into parameters such as discriminatory index (DI), typeability, and ISD of the isolates. Comparing these methods enables researchers to select the most appropriate technique [15, 34, 49].

RAPD detects genetic variation by randomly amplifying segments of target DNA with a short oligonucleotide random sequence [15, 50]. In this study, two primers of RAPD have been used for the characterization of 60 *P. mirabilis* isolates. The two primers gave low typeability% (46.67% and 48.33%) and moderate ISD% (46.67% and 43.33%). In addition, they gave total bands of 9 and 6 bands with %MB equal 66.7% and PB% equal to 33.3%. when investigating the discrimination index, they showed low SID (0.72 and 0.735) below the acceptable level (0.95). OPZ19 classified the isolates into 28 clusters at 70, 80 and 80% cutoff, while OPX13 classified them into 20 clusters at 70% cutoff. In contrary [14], showed that RAPD markers had high discrimination index such as *OPA19* (SID 0.847), and *OPX13* (SID 0.921). Regarding that Three primers used *(OPA11, OPX13 and OPZ8)* provided a high level of discrimination among all *P. mirabilis* isolates. The effectiveness of RAPD markers for *Proteus* fingerprinting has been reported previously [51]. The relatively low reproducibility of RAPD typing limited its application to large-scale in laboratory studies.

Four intergenic single sequence repeats (ISSR) primers were used in this study. The best marker used was ISSR 2 where its typeability was 90%, ISD% was 68.33 and gave 38 clusters at 90% cutoff. Its MP% and PM% were equal to 50% with EMR = 1.5. The SID was high (0.983) with the highest number of patterns (57). The other primers were different in efficiency. ISSR1 was the best in typeability (85%) and SID (0.955). While ISSR6 was best at PM% (60%), ISD% (60%), SID (0.934), gave 36 clusters at 90% cutoff with 36 pattern and EMR = 1.8. ISSR 3 was the best marker showing the similarity between isolates as its ISD% (23.33), typeability (55%), number of clusters at 90% cutoff was 14 and its SID was 0.776. A study conducted by Michelim et al*.* showed ISSR primers gave a higher number of bands with PM% = 77.5%. In contrast to our result, the best marker was ISSR 6 as its SID was 1 followed by ISSR 1 and ISSR 2. ISSR 3 was the last one in discrimination between isolates as its SID was 0.458 [14].

ERIC-PCR typing method gave the highest efficiency in all aspects used as typeability (90 and 98.33%), ISD% (85 and 95%), number of clusters at 90% cutoff (51 and 57), PM% (77.77 and 66.67%), EMR (5.4 and 5.3), and SID (0.989 and 0.999). So, it was the best method used for the discrimination of isolates. ERIC-PCR gave PM% equal 90% and SID = 0.97 in a study of [14].The study of [52] showed that ERIC-PCR classified 98 *E. coli* isolates into six clusters proving that ERIC-PCR had high discriminatory power in genotyping strains. [53] reported that ERIC-PCR completely typed the 60 *P. mirabilis* isolates with high reproducibility. In addition, ERIC-PCR was reported as the best method for discrimination of *P. aeruginosa* isolates as compared to other PCR-based typing methods[15].

In this study, Box-PCR gave 70% for typeability, 63.3% for ISD%, 55. 56% for PM%, 0.911 for SID and 38 clusters at 90% cutoff. ERIC-PCR and BOX markers allowed separate the 29 *Proteus* isolates into several groups [14]. BOX-PCR and ERIC-PCR gave highly comparable efficiency in evaluating the genetic relatedness of 29 pathogenic *Leptospira* strains representing higher discriminatory index using ERIC-PCR 0.826 than BOX-PCR 0.809 [54].

REP-PCR in this study gave high typeability % (88.3 and 98.3%), PM% (57.14 and 60%) and SID (0.985 and 0.84). Although *REP-PCR 21R* has a high percent of typeability (98.33%) similar to the typeability percent of *ERIC2* but has the lowest ISD (25%) value compared by different typing methods as ERIC and REP-PCR. So, the Index of Strain Diversity percentage gave more accurate results for the comparison of different typing methods. Twenty-nine *P. mirabilis*, showed that all 29 *P. mirabilis* isolates used were separated in various groups by using BOX-PCR and ERIC-PCR. This study shows resemble results with all primers used except for REP-PCR1R and 2I primers where they showed low discriminatory power (SID = 0.621).

By comparing the five different PCR-based typing method, ERIC was the best typing method in all of the studied parameters followed by REP-PCR, BOX-PCR, ISSR then at last RAPD-PCR which gave lower results than other methods. [14] reported that RAPD, ERIC-PCR and BOX-PCR markers with a high discriminatory power for typing clinical *P. mirabilis* isolates. ERIC-PCR is a cheap, easy-to-be-done, reliable and rapid method besides its high discrimination ability [15].

The probability that pairs of isolates assigned to the same type by one typing method are also typed as identical by the other is indicative of the congruence between typing methods. The congruence obtained by Rand’s coefficient indicated that ISSR2 was best predicted by ERICIR, ISSR1, ISSR2, ISSR6 and ERIC 1R were best predicted by ERIC 2 and ISSR2, ERIC1R and ERIC2 were best predicted REP-PCR 1R. the evaluation of the congruence using Wallace coefficient revealed that REP-PCR 21R and ERIC1R, OPZ19, ISSR2, ISSR6, REP-PCR1R, REP-PCR21R and ERIC2 showed moderate congruence (0.579 and o.5, respectively). While a complete congruence between ISSR3 and ERIC2 was found (W = 1). No or poor congruence occurred with the other typing primers signifying that they clustered the isolates in different methods.

In conclusion; we evaluated the antibiotic resistance, the virulence factors, biofilm production and the molecular typing methods for the differentiation of *P. mirabilis* isolates. The phenotypic methods used couldn’t discriminate between isolates. The best discrimination was achieved by PCR-based typing methods (ERIC-PCR, BOX-PCR, REP-PCR, RAPD and ISSR). ERIC-PCR was the best method that differentiated *P. mirabilis* isolates followed by REP-PCR. The other methods gave medium to high discriminatory efficiency in *P. mirabilis*. In addition, by evaluating the congruence between typing methods, ERIC-PCR was the best method giving high values for Rands and Wallace coefficients with other typing methods.

Thus; the ERIC-PCR method is recommended for use in future epidemiological studies of clinical isolates from various microorganisms isolated from infectious diseases.

## Supplementary Information

Additional file 1.

## Data Availability

No datasets were generated or analysed during the current study.
