# Supplementary material for: Assessment of typing methods, virulence genes profile and antimicrobial susceptibility for clinical isolates of Proteus mirabilis
Source: Ann Clin Microbiol Antimicrob. 2025 Jan 15;24:4. doi: 10.1186/s12941-024-00770-8 (PMC11734338; doi:10.1186/s12941-024-00770-8)
Supplement: Supplementary file 1 — Additional file 1. [file 12941_2024_770_MOESM1_ESM.docx]

**Supplementary table (1) The characteristics of the *Proteus mirabilis* isolates in relation collection data.**

| **Isolate No.** | **Sex** | **Age** | **Clinical case** | **Date of isolation** |
| --- | --- | --- | --- | --- |
| **1** | **M** | **23** | **Contracted Bladder Radiation therapy** | **6/8/2016** |
| **2** | **M** | **52** | **Renal Stone** | **7/8/2016** |
| **3** | **M** | **2** | **Meatus** | **7/8/2016** |
| **4** | **M** | **13** | **Hypospadias** | **8/8/2016** |
| **5** | **M** | **60** | **Renal Stone** | **9/8/2016** |
| **6** | **M** | **2** | **Hypospadias** | **12/8/2016** |
| **7** | **M** | **80** | **Transurethral resection of prostate** | **21/8/2016** |
| **8** | **F** | **9** | **Post Repair of Vesicocutaneous Fistula** | **3/9/2016** |
| **9** | **F** | **29** | **Renal Stone** | **3/9/2016** |
| **10** | **M** | **65** | **Renal Stone** | **3/9/2016** |
| **11** | **M** | **16** | **Recurrent Structure, Posterior Urethra** | **2/10/2016** |
| **12** | **M** | **48** | **Renal Urethroplasty** | **9/10/2016** |
| **13** | **M** | **2** | **Inguinal Hernia** | **15/10//2016** |
| **14** | **M** | **19** | **Recurrent Structure, Anterior Urethra** | **1/11/2016** |
| **15** | **M** | **67** | **Urine Retention** | **2/11/2016** |
| **16** | **M** | **18** | **Post Bladder Exstrophy Repair** | **9/11/2016** |
| **17** | **M** | **73** | **Post Simple Cystectomy** | **9/11/2016** |
| **18** | **M** | **21** | **Total Urinary Incontinence** | **12/11/2016** |
| **19** | **F** | **9** | **Bladder neck reconstruction** | **12/11/2016** |
| **20** | **M** | **8** | **Neuropathic Bladder** | **19/11/2016** |
| **21** | **M** | **13** | **Total Urinary Incontinence** | **19/11/2016** |
| **22** | **M** | **83** | **Bladder Tumor** | **22/11/2016** |
| **23** | **F** | **30** | **Nephrotic Syndrome** | **22/11/2016** |
| **24** | **M** | **14** | **Smooth Sphincter** | **6/12/2016** |
| **25** | **F** | **5** | **Congenital adrenal hyperplasia** | **9/12/2016** |
| **26** | **M** | **31** | **Renal Stone** | **14/12/2016** |
| **27** | **M** | **84** | **Stone Posterior Urethra** | **19/12/2016** |
| **28** | **M** | **67** | **Transurethral resection of prostate** | **25/12/2016** |
| **29** | **M** | **8** | **Hypospadias** | **29/12/2016** |
| **30** | **F** | **54** | **Repair of Vesicovaginal Fistula** | **13/1/2017** |
| **31** | **F** | **33** | **Tension free vaginal tape** | **15/1/2017** |
| **32** | **M** | **60** | **Renal Stone** | **17/1/2017** |
| **33** | **M** | **67** | **Obstructed Benign prostatic hyperplasia** | **31/1/2017** |
| **34** | **M** | **7** | **Hypospadias** | **1/2/2017** |
| **35** | **M** | **88** | **Bladder Tumor** | **2/2/2017** |
| **36** | **F** | **22** | **Renal Stone** | **4/2/2017** |
| **37** | **F** | **52** | **Renal Stone** | **9/2/2017** |
| **38** | **M** | **8** | **Hypospadias** | **12/2/2017** |
| **39** | **M** | **5** | **Hypospadias** | **15/2/2017** |
| **40** | **M** | **85** | **Transurethral resection of prostate** | **15/2/2017** |
| **41** | **M** | **67** | **Chronic kidney disease** | **16/2/2017** |
| **42** | **F** | **25** | **Urethral Tube multiple Vesical Stones** | **17/2/2017** |
| **43** | **M** | **46** | **Metastasis** | **17/2/2017** |
| **44** | **M** | **30** | **Chronic kidney disease** | **17/2/2017** |
| **45** | **M** | **5** | **Bilateral Chronic Pyelonephritis** | **19/2/2017** |
| **46** | **F** | **21** | **Renal Stone** | **19/2/2017** |
| **47** | **M** | **62** | **Metastatic Prostatic carcinoma** | **19/2/2017** |
| **48** | **M** | **4** | **Post Bladder Exstrophy Repair** | **7/3/2017** |
| **49** | **M** | **3** | **Hypospadias** | **13/3/2017** |
| **50** | **F** | **41** | **Renal Stone** | **14/3/2017** |
| **51** | **M** | **57** | **Post traumatic Posterior Urethral Structure** | **17/3/2017** |
| **52** | **M** | **75** | **Grade Bladder Transitional cell carcinoma** | **21/3/2017** |
| **53** | **F** | **8** | **Post Repair of Vesicocutaneous Fistula** | **25/3/2017** |
| **54** | **F** | **12** | **Post Repair of Vesicocutaneous Fistula** | **26/3/2017** |
| **55** | **F** | **6** | **Post Repair of Vesicocutaneous Fistula** | **28/3/2017** |
| **56** | **M** | **52** | **Recurrent Bone marrow Urethral Structure** | **4/4/2017** |
| **57** | **F** | **2** | **Right Pyeloplasty** | **4/4/2017** |
| **58** | **F** | **16** | **Total Urinary Incontinence** | **8/4/2017** |
| **59** | **M** | **5** | **Hypospadias** | **8/4/2017** |
| **60** | **M** | **56** | **Staghorn Stone** | **15/4/2017** |

**M: Male F: Female**
